# Synergies between environmental degradation and climate variation on malaria re-emergence in southern Venezuela: a spatiotemporal modelling study

**DOI:** 10.1016/S2542-5196(22)00192-9

**Published:** 2022-09

**Authors:** Isabel K Fletcher, Maria Eugenia Grillet, Jorge E Moreno, Chris Drakeley, Juan Hernández-Villena, Kate E Jones, Rachel Lowe

**Affiliations:** Centre for Mathematical Modelling of Infectious Diseases, London School of Hygiene & Tropical Medicine, London, UK; Centre on Climate Change and Planetary Health, London School of Hygiene & Tropical Medicine, London, UK; Instituto de Zoología y Ecología Tropical, Facultad de Ciencias, Universidad Central de Venezuela, Caracas, Venezuela; Centro de Investigaciones Francesco Vitanza, Servicio Autónomo Instituto de Altos Estudios Dr Arnoldo Gabaldon, Ministerio del Poder Popular para la Salud, Bolívar, Venezuela; Department of Infection Biology, London School of Hygiene & Tropical Medicine, London, UK; Instituto de Zoología y Ecología Tropical, Facultad de Ciencias, Universidad Central de Venezuela, Caracas, Venezuela; Centre for Biodiversity and Environment Research, University College London, London, UK; Centre for Mathematical Modelling of Infectious Diseases, London School of Hygiene & Tropical Medicine, London, UK; Centre on Climate Change and Planetary Health, London School of Hygiene & Tropical Medicine, London, UK; Barcelona Supercomputing Center, Barcelona, Spain; Catalan Institution for Research and Advanced Studies, Barcelona, Spain

## Abstract

**Background:**

Environmental degradation facilitates the emergence of vector-borne diseases, such as malaria, through changes in the ecological landscape that increase human–vector contacts and that expand vector habitats. However, the modifying effects of environmental degradation on climate–disease relationships have not been well explored. Here, we investigate the rapid re-emergence of malaria in a transmission hotspot in southern Venezuela and explore the synergistic effects of environmental degradation, specifically gold-mining activity, and climate variation.

**Methods:**

In this spatiotemporal modelling study of the 46 parishes of the state of Bolívar, southeast Venezuela, we used data from the Venezuelan Ministry of Health including population data and monthly cases of *Plasmodium falciparum* malaria and *Plasmodium vivax* malaria between 1996 and 2016. We estimated mean precipitation and temperature using the ERA5-Land dataset and used monthly anomalies in sea-surface temperature as an indicator of El Niño events between 1996 and 2016. The location of suspected mining sites in Bolívar in 2009, 2017, and 2018 were sourced from the Amazon Geo-Referenced Socio-Environmental Information Network. We estimated measures of cumulative forest loss and urban development by km^2^ using annual land cover maps from the European Space Agency Climate Change Initiative between 1996 and 2016. We modelled monthly cases of *P falciparum* and *P vivax* malaria using a Bayesian hierarchical mixed model framework. We quantified the variation explained by mining activity before exploring the modifying effects of environmental degradation on climate–malaria relationships.

**Findings:**

We observed a 27% reduction in the additional unexplained spatial variation in incidence of *P falciparum* malaria and a 23% reduction in *P vivax* malaria when mining was included in our models. The effect of temperature on malaria was greater in high mining areas than low mining areas, and the *P falciparum* malaria effect size at temperatures of 26·5°C (2·4 cases per 1000 people [95% CI 1·78–3·06]) was twice as high as the effect in low mining areas (1 case per 1000 people [0·68–1·49]).

**Interpretation:**

We show that mining activity in southern Venezuela is associated with hotspots of malaria transmission. Increased temperatures exacerbated malaria transmission in mining areas, highlighting the need to consider how environmental degradation modulates climate effect on disease risk, which is especially important in areas subjected to rapidly rising temperatures and land-use change globally. Our findings have implications for the progress towards malaria elimination in the Latin American region. Our findings are also important for effectively targeting timely treatment programmes and vector-control activities in mining areas with high rates of malaria transmission.

**Funding:**

Biotechnology and Biological Sciences Research Council, Royal Society, US National Institutes of Health, and Global Challenges Research Fund.

## Introduction

Land-use change, climate variability, and climate change are strongly implicated in driving the emergence and re-emergence of infectious diseases.^1^ Environmental degradation, including deforestation for agriculture or mining activities, can compromise human health by modifying the natural reservoir of disease pathogens and vector habitats, thereby facilitating increases in human exposure to zoonoses. Malaria is a vector-borne disease that is sensitive to environmental conditions^2^ and that imposes widespread global disease burden, with an estimated 241 million cases recorded in 2020.^3^ The links between climate and malaria in endemic regions of the world are well established; for example, seasonal increases in rainfall and temperatures are linked to the malaria transmission season across sub-Saharan Africa^4^ because these conditions are favourable for the development of the *Anopheles* mosquito vector and *Plasmodium* malaria parasite.^5^

In addition to climate variation, land-use changes can also affect malaria transmission; they do so predominantly through the alteration of *Anopheles* vector ecology.^6^ In the Peruvian Amazon, ecological fragmentation resulting from deforestation favours the primary malaria vector *Anopheles darlingi.* In deforested areas, the availability of *A darlingi* breeding habitats, which are characterised by forest edges and bodies of water in permanent sunlight, is increased.^6^ The relationship between deforestation and malaria, however, is highly context-dependent and subject to strong socioeconomic feedbacks, such as socioeconomic development, which can reduce the effect of mosquito breeding habitats on malaria risk.^7^ Understanding the effect of land-use change on disease risk, alongside other important factors including climate, will be required to manage landscapes that are increasingly dominated by human activities.^8^ There has been little exploration of how land use modulates the effect of climate variation. Microclimatic changes associated with environmental disturbances such as forest clearance result in increased temperatures, subsequently enhancing mosquito survivorship and vectorial capacity.^9,10^ Consequently, it is expected that land-use change could alter the effect of climate variation on disease transmission. These modifying effects are important to consider given the global increases in land-use changes, climate variation, and climate change, as well as recent plateaus in the global malaria response.^11^

An assessment of the interacting effects of land use and climate on malaria is of notable concern in politically unstable regions that have uncontrolled surges in disease transmission. In this study, we used southern Venezuela as a case study because the current epidemic growth in malaria cases is very high.^11^ This worrying epidemic is a regional problem that threatens to reverse malaria elimination progress across the Latin American region.^12^ Malaria transmission in Venezuela is thought to be sustained by disease hotspots in southern areas that have been degraded by mining activity (primarily for gold); these areas have expanded since the 2010s due to political instability and economic collapse.^13^ The clustering of malaria cases in mining areas creates source–sink dynamics across Venezuela, bolstering hotspots of transmission in the south of the country.^13^ High entomological inoculation rates (number of infective bites) and abundance of *A darlingi* identified in gold-mining areas in Venezuela^14,15^ allow for high exposure of vulnerable migrant populations to malaria transmission.^16^ Further work has indirectly linked increasing rates of malaria to mining activity, not only in Venezuela. Increased densities of anopheline vectors documented in illegal gold-mining sites in French Guiana have subsequently been linked to malaria outbreaks.^17^ Additionally, studies in Peru and Brazil, and recently Guyana, have linked gold prices^18,19^ and rates of gold production to malaria.^20,21^ In Colombia, 32% of national malaria cases were from gold-mining areas.^21^ In Suriname, the mobility of mining workers was associated with an increase in the number of imported malaria cases.^22^ Gold-mining areas are important reservoirs of malaria transmission that often go undetected due to poor surveillance and frequent use of improper self-medication to treat malaria symptoms.^23^ Despite these findings, previous research has not yet been able to draw a direct link between mining activity and malaria, and it is unclear to what extent mining activity can explain patterns of malaria incidence. Moreover, previous research has overlooked the effect of climate variation on malaria, considering only the effect of mining on malaria independently of other important environmental factors. Little attention has been placed on how environmental degradation caused by mining activity interacts with, and modulates, the effect of climate on malaria risk. Despite local studies that provide evidence of how hotspots of malaria transmission are maintained in mining areas, malaria transmission rates in Venezuela continue to rise,^11^ highlighting the need for a more comprehensive understanding of the spatiotemporal drivers of transmission. In this study, we explore the spatiotemporal drivers of malaria re-emergence in southern Venezuela, investigating the influence of environmental degradation, specifically mining activity, and its modifying effect on the relationship between climate and malaria.

## Methods

### Study area and data sources

**Figure 1:**
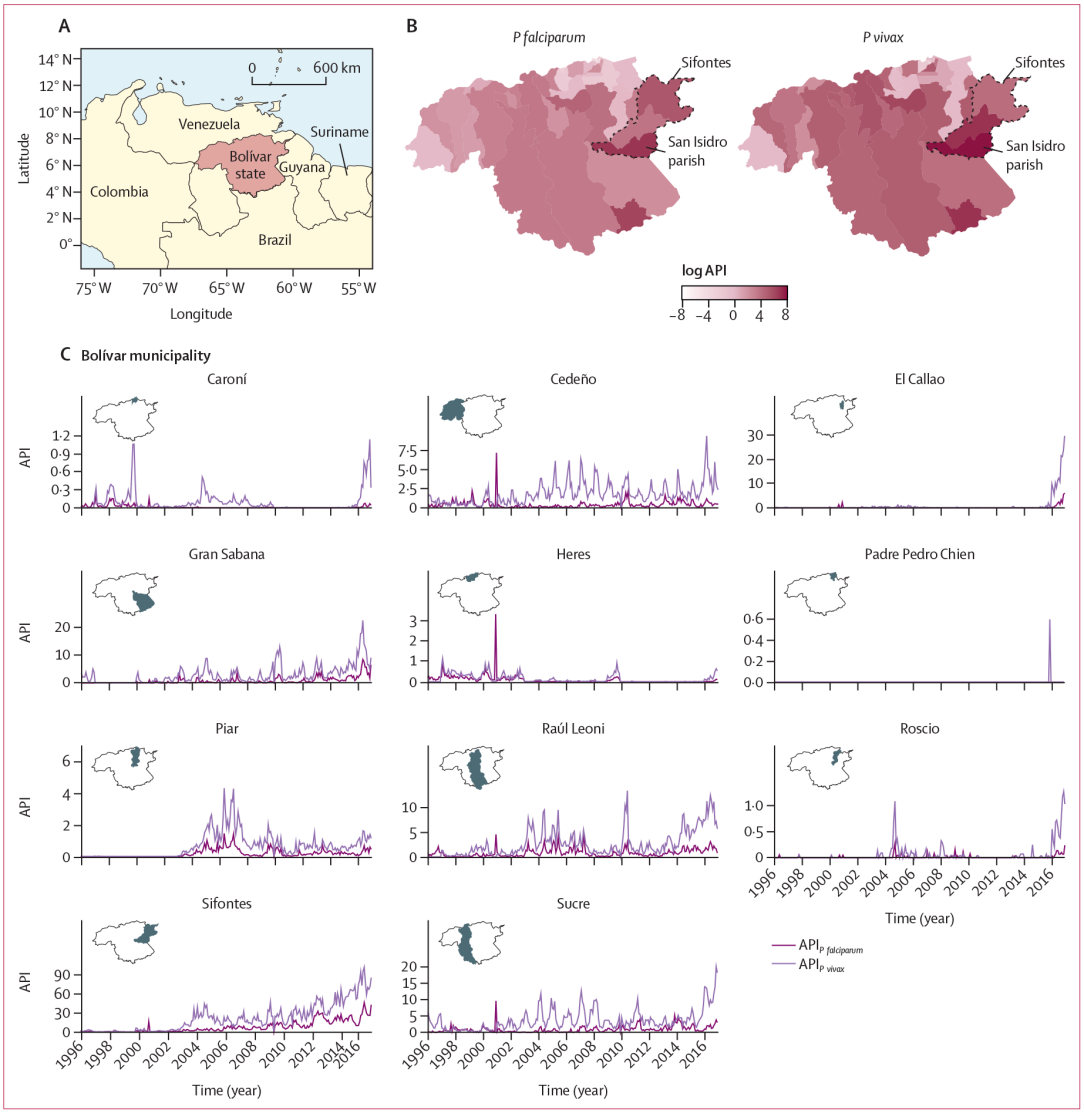
Geographical context and spatiotemporal trends of malaria incidence in Bolívar 1996–2016 A) Location of the state of Bolívar in southern Venezuela. B) API per 1000 people, log transformed, of Plasmodium *falciparum* and *Plasmodium vivax* malaria across Bolívar in 2016. C) API per 1000 people of *P falciparum* (dark purple) and *P vivax* (light purple) malaria in the 11 municipalities of Bolívar between 1996 and 2016. Inset maps show locations of each municipality in Bolívar (grey shading). API=annual parasite incidence.

This spatiotemporal modelling study of Venezuela is focused specifically on the state of Bolívar, which is located in south-eastern Venezuela and shares borders with Guyana and Brazil. Bolívar has a total area of 240 500 km^2^, had a total population in 2018 of 1837 485,^13^ and is subdivided into 11 municipalities that are further divided into 46 parishes (third-level administrative units; appendix p 3). Although malaria was eliminated in approximately 75% of Venezuelan territory in the 1960s, transmission of *Plasmodium falciparum* and *Plasmodium vivax* has persisted in remote southern regions of the country, including Bolívar, and accounted for 47% of national cases in 2017.^13^ From 2014 onwards, local malaria transmission has re-emerged in new areas of the country, including in the southwest. This re-emergence has been exacerbated by the political, humanitarian, and health crises in the country, which have had a damaging effect on vector control, disease surveillance, and access to treatment. Malaria transmission in Bolívar is highly focal; for example, the municipality of Sifontes in the northeast has had local clustering of malaria cases that have sustained high transmission (figure 1).^13^ The majority of malaria infections in Bolívar result from *P vivax* (70–80%) and *P falciparum* (20–30%) parasites and the most common malaria vectors in Bolívar are *A darlingi* and *Anopheles albitarsis* sensu lato.^13^

The number of monthly cases of *P falciparum* and *P vivax* malaria reported at local health centres and confirmed by blood smears for each of the 46 parishes in Bolívar from 1996 to 2016 by were provided for the Ministry of Health. Annual population estimates per 1000 people per parish in the same period were also sourced from the National Statistics Office of Venezuela. Monthly estimates of total precipitation and mean temperature for each parish in Bolívar in the same period were obtained from the ERA5-Land dataset.^24^ Using data from the National Oceanic and Atmospheric Administration, we collected monthly anomalies in sea-surface temperatures in the Niño 3.4 index region, as an indicator of the El Niño Southern Oscillation phenomenon.^25^

The location of suspected mining sites in Bolívar (n=2460) were sourced from the Amazon Geo-Referenced Socio-Environmental Information Network (appendix p 4). This dataset consists primarily of sites for gold extraction, which is the main economic activity in the area. The dataset also contains sites in which other resources such as bauxite, aluminium, calcite, and iron are extracted. Locations of sites from satellite images were captured in 2009, 2017, and 2018 and some smaller sites identified by satellite imagery were verified by local communities. We counted the total number of mining sites per parish and included all sites in our dataset. We included sites that were labelled as inactive, under the assumption that the habitat created by land clearance for mines including pools of stagnant water can remain once mining pits are abandoned. We estimated measures of cumulative forest loss in km^2^ and urban development in km^2^ for each parish from 1996 to 2016 (appendix p 2) using annual land cover maps from the European Space Agency Climate Change Initiative .

### Spatiotemporal modelling of *P falciparum* and *P vivax* malaria incidence

To investigate drivers of malaria re-emergence in Bolívar, we formulated spatiotemporal Bayesian hierarchical mixed models to model monthly incidence of *P falciparum* and *P vivax* malaria between 1996 and 2016. We constructed separate models for each malaria parasite to account for differences between parasites, which include their extrinsic incubation period, the ability to cause relapsing infections, the effectiveness of vector control, and sensitivity to local climate conditions.^26,27^ A zero-inflated negative binomial (ZINegBin) model was used to allow for excess zeros present in parishes of Bolívar with low malaria incidence, which are not explained by a standard negative binomial distribution. *P falciparum* and *P vivax* malaria cases ($y_{\text{st}}$) were assumed to follow a negative binomial distribution, with overdispersion parameter (κ; equation 1). We modelled the mean ($\mu_{\text{st}}$) number of monthly (t=1 to 252) malaria cases for 21 years (1996–2016) in each parish (s=1 to 46), for each malaria parasite in Bolívar as the log population ($P_{\text{st}}$) included as an offset, and the annual parasite incidence (API; the number of malaria cases per 1000 individuals per month for *P falciparum* and *P vivax* malaria), API ($\log(\rho_{\text{st}})$; equation 2). The API, $\log(\rho_{\text{st}})$, was then estimated as a combination of spatiotemporal covariates, $\sum \beta_{i}x_{\text{ist}}$, for land use (mining, deforestation, and urbanisation), climate (mean temperature and total precipitation), and spatial and temporal random effects, in which α is the model intercept (equation 3).


(1) $$y_{\mathrm{st}}∼\text{ZINegBin}(\mu_{\text{st}},\kappa )$$

(2) $$\log(\mu_{\text{st}})=\log(P_{\text{st}})+\log(\rho_{\text{st}})$$

(3) $$\log(\rho_{\text{st}})=\alpha +\sum \beta_{i}x_{\text{ist}}+m_{t}+a_{t}+\upsilon_{s}+v_{s}$$

We included spatiotemporal random effects in the model framework to account for unobserved confounding factors and to capture unknown variability in the data. Unexplained variation might be due to unmeasured factors, such as vector control and population movements. Random effects for each month were specified in our model ($m_{t}$), by introducing a first-order random walk latent model, which allowed for malaria incidence in one month to depend on incidence in the previous month; to capture any seasonality in malaria incidence in Bolívar. Exchangeable random effects were specified for each year ($a_{t}$, 1996–2016) to allow for additional sources of variability that could not be captured by the model covariates.^28^ To allow for spatial correlation in malaria incidence across parishes in Bolívar, we assigned conditional intrinsic Gaussian autoregressive model priors on the spatial random effects ($\upsilon_{s}$). We also specified independent diffuse Gaussian exchange priors ($v_{s}$) for each parish in order to account for any additional uncorrelated variation in malaria incidence across parishes in Bolívar that could not be measured.^28,29^

### Model implementation

We formulated spatiotemporal models of *P falciparum* and *P vivax* malaria. Our model covariates were monthly anomalies in sea-surface temperatures in the Niño 3·4 index region, mining (continuous variable [number of mines per parish]), deforestation (measured as cumulative forest loss between 1996 and 2016), urban development (measured as cumulative urban increase between 1996 and 2016), and non-linear temperature and rainfall. All covariates in our models were scaled by subtracting the covariate mean from each value and dividing by the covariate standard deviation. We investigated how land-use changes modify the effect of local climate conditions on malaria incidence in Bolívar by including an interaction term between mining activity (included as a categorical variable; low [≤2 mines]) *vs* high [>2 mines]) and each climate variable (temperature and precipitation, specified as a non-linear term [appendix p 2]). We classified high mining activity as areas in Bolívar with more than two mines (the median for the dataset) and low levels as two or less mines (appendix p 4). The minimum number of mines per parish was zero and the maximum was 446. Model fit was assessed using Bayesian methods of model comparison, the deviance information criterion,^30^ and Watanabe-Akaike information criterion.^31^ Models were implemented using integrated nested laplace approximation, which, in contrast to Markov Chain Monte Carlo methods, uses numerical approximations of model parameters and is computationally more efficient and a faster alternative for spatiotemporal disease modelling.^32^

### Role of the funding source

The funders of the study had no role in study design, data collection, data analysis, data interpretation, or writing of the report.

## Results

Malaria transmission in Bolívar (figure 1A) is highly focal. The majority of malaria incidence in 2016 was concentrated in the north-east of the region, in Sifontes (figure 1B). Between 1996 and 2016, a total of 455 461 *P vivax* and 148 169 *P falciparum* malaria cases were recorded in Bolívar, with cases of *P falciparum* increasing from 1535 in 1996 to 26 232 in 2016 (a 1609% increase) and with cases of *P vivax* increasing from 2920 cases in 1996 to 90 106 cases in 2016 (a 2986% increase). The increase of malaria cases in Bolívar was driven mainly by transmission patterns in the municipality of Sifontes (San Isidro, Dalla Costa, and Tumeremo parishes; appendix p 3); a known transmission hotspot (figure 1C). Incidence in Sifontes has been increasing since 2005 (figure 1C), and 254774 (56%) of the 455461 total *P vivax* cases and 93 752 (63%) of the 148 169 total *P falciparum* cases between 1996 and 2016 were recorded in this municipality. The highest API of *P vivax* malaria was 3198 cases per 1000 people in San Isidro parish in 2016 (figure 1B). 1074 cases per 1000 people of *P falciparum* malaria was recorded in San Isidro parish in the same year. Malaria re-emergence in Bolívar is heterogeneous; for some municipalities in Bolívar, such as Sucre and Cedeño, incidence has remained high since the early 2000s, while others such as Raúl Leoni have had a sharp increase in malaria incidence since 2013 (figure 1C).

**Figure 2:**
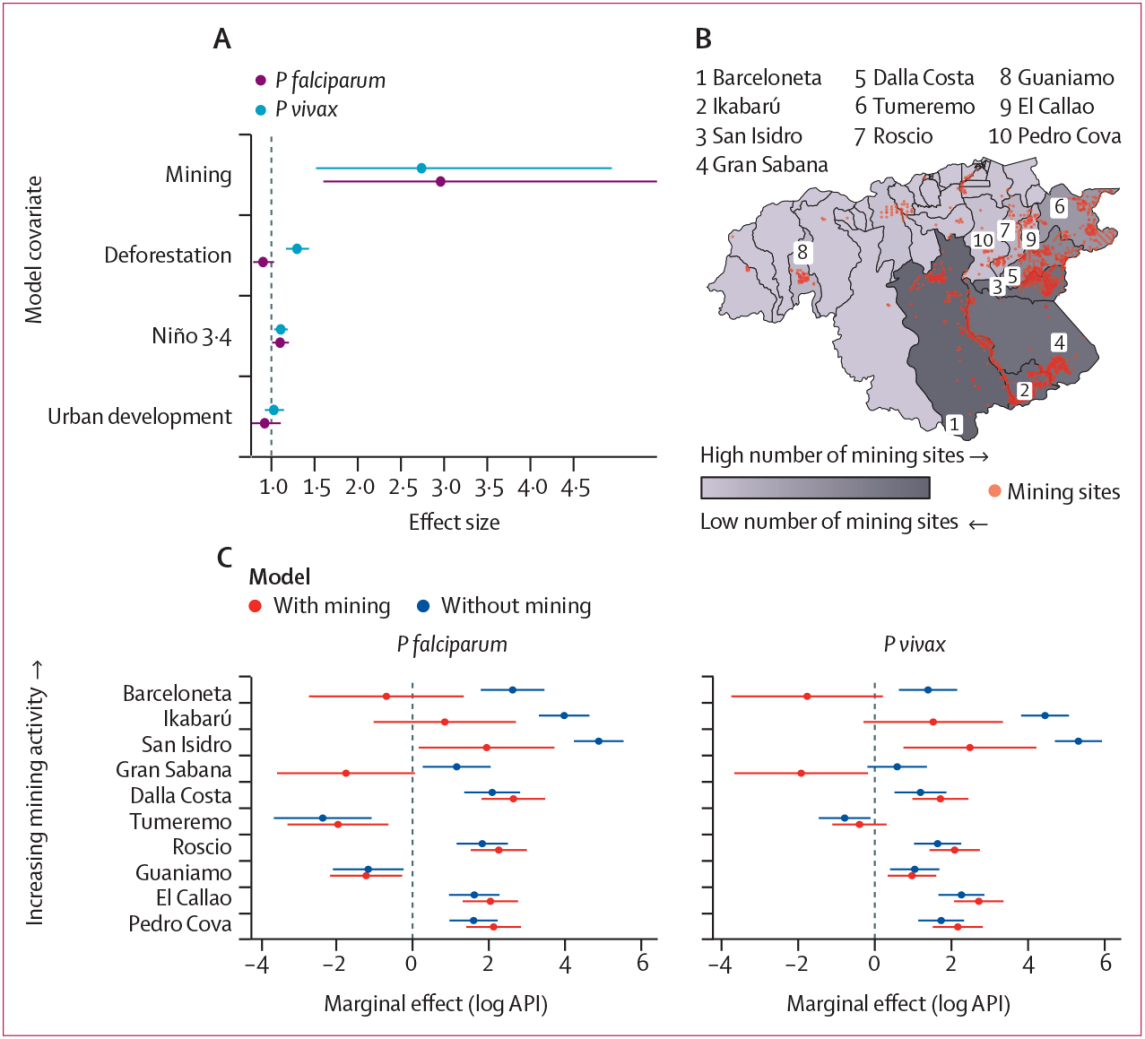
Environmental and socioeconomic drivers of malaria in Bolívar (A) Effect size and 95% CIs for spatiotemporal models of *Plasmodium falciparum* malaria (purple bars) and *Plasmodium vivax* malaria (blue bars) incidence. The model included an interaction term between levels of mining (high [>2 mines] and low [≤2 mines]) and non-linear functions of temperature and rainfall. The model also included random effects to account for seasonality, interannual variability, and spatial dependency structures. (B) Locations of mining sites (red dots) in Bolívar identified through remote sensing and total number of mining sites per parish. Dark grey colours show parishes with a high number of mining sites and light grey colours represent parishes with few or no mining sites. Labels are shown for the ten parishes with the highest mining activity. (C) Variation in malaria incidence explained by mining activity. Marginal effect (mean and 95% CIs of the spatial random effect) of log API, of spatiotemporal models for *P falciparum* and *P vivax* malaria that exclude (blue) and include (red) mining activity across Bolívar as a covariate. A reduction in mean estimate towards zero indicates areas in which mining activity explains the spatial variation in malaria incidence. Estimates are shown for the ten parishes in Bolívar with the highest number of mines. The model also includes linear effects of mining, deforestation, urban development, sea-surface temperature anomalies characteristic of El Niño Southern Oscillation events, an interaction term between high and low levels of mining and non-linear functions of temperature and rainfall, as well as random effects to account for seasonality, interannual variability, and spatial dependency structures. API=annual parasite incidence.

We found extensive forest loss across Bolívar, with most deforestation taking place between 1997 and 2004 (appendix p 5); during this timeframe, we also observed peaks in malaria incidence in Caroní, Cedeño, and Heres municipalities (figure 1C). We found that areas in Bolívar with higher malaria incidence had greater forest loss than areas with lower incidence (appendix p 5). For instance, Barceloneta parish, which had an API of 152 *P vivax* cases per 1000 people in 2016 had a forest loss of 1340 km^2^ in 2016. Comparatively, Gran Sabana parish, which had an API of 62 malaria *P vivax* cases in 2016, had a forest loss of 1024 km^2^; 24% less than the forest loss in Barceloneta. Deforestation was associated with increases in *P vivax* malaria (effect size of 1·3 cases per 1000 people [95% CI 1·2–1·4]) but not *P falciparum* malaria (effect size 0·9 cases per 1000 people [95% CI 0·8–1·0]; (figure 2A). Positive anomalies of the Niño 3·4 index in Bolívar, which represent higher than average sea temperatures in the Niño 3·4 region and are associated with warmer temperatures and drought conditions in central and eastern Bolívar (appendix p 7), were associated with slight increases in both *P falciparum* malaria (1·1 cases per 1000 [95% CI 1·0–1·2]) and *P vivax* malaria (1·1 cases per 1000 [95% CI 1·0–1·2]; figure 2A). There was no significant association between malaria incidence and urban development with either *P falciparum* (effect size 0·9 [95% CI 0·8–1·1]) or *P vivax* effect size 1·0 [95% CI 0·9–1·1]).

2460 mines were identified in Bolívar, representing 96% of the 2561 total mines identified in the whole of Venezuela (figure 2B; appendix p 4). In our model, we identified a strong positive association between mining activity and both *P falciparum* malaria (effect size of 3·0 cases per 1000 people [95% CI 1·6–5·5]) and *P vivax* malaria (2·7 cases per 1000 people [1·5 - 4·9]; figure 2A). We determined the relative effect of mining activity on malaria transmission in Bolívar by comparing the spatial random effects of a model in which we explicitly accounted for mining activity, with a model in which we did not (figure 2C; appendix p 8). For visualisation purposes, we looked at the difference in the marginal effect of these models in the ten parishes in Bolívar with the highest mining activity. These parishes had a reduction in the value of the marginal effect (log API) towards zero when mining activity was explicitly accounted for, indicating that mining accounted for a large proportion of unexplained variation (figure 2C). The marginal effects shrank in 25 (54%) of the 46 parishes in Bolívar for *P falciparum* models and in 21 (46%) parishes for *P vivax* models (appendix p 8). In a few parishes, such as Guaniamo and El Callao, the marginal effect did not change considerably (appendix p 8). In other parishes, such as such as Aripao and Dalla Costa, the magnitude of the marginal effect increased with the addition of mining activity, indicating that unobserved factors contribute to additional variation in malaria incidence. Overall, when mining was included in the models, there was a reduction in the additional unexplained spatial variation of *P falciparum* incidence by 27% and by 23% for *P vivax* incidence, indicating that mining accounted for some of the spatial variation in malaria incidence.

**Figure 3:**
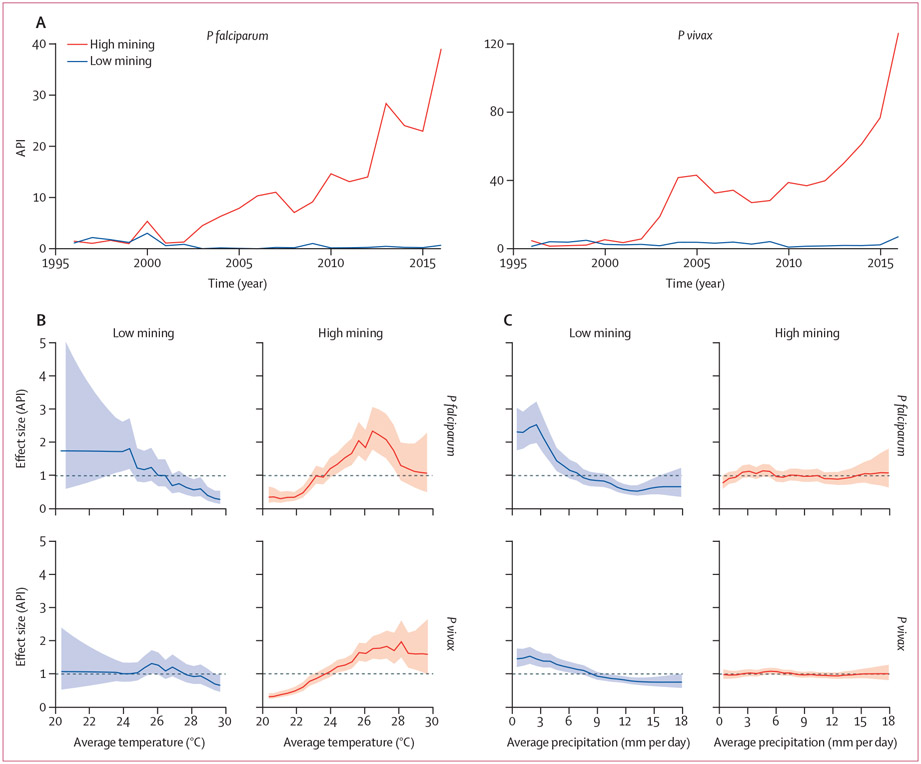
Combined effect of mining activity and climate variation on malaria risk in Bolívar Distribution (median, upper, and lower quartiles) of API, log transformed in 2016, of *P falciparum* and *P vivax* malaria in areas of Bolívar with low and high mining activity (A). Effect size (API; solid line) and 95% CIs (shading) for the relationship between mean temperature (B) and precipitation (C) and *P falciparum* and *P vivax* incidence in areas of low and high mining activity. The model included an interaction term between high and low levels of mining and non-linear functions of temperature and rainfall. The model also included linear effects of mining, deforestation, urban development, sea-surface temperature anomalies characteristic of El Niño Southern Oscillation events, as well as random effects, to account for seasonality, interannual variability, and spatial dependency structures. API=annual parasite incidence.

Parishes of Bolívar that were classed as having high mining activity (>2 mines) recorded a total API in 2016 of 38·8 *P falciparum* malaria cases per 1000 people and 126 *P vivax* malaria cases per 1000 people, compared with 0·8 *P falciparum* malaria cases per 1000 people and 7 *P vivax* malaria cases per 1000 people in areas with low mining activity (≤2 mines; figure 3A). We assessed the effect of climate on malaria incidence in areas of high mining activity compared with areas of low mining activity by specifying an interaction term between mining activity and each non-linear climate variable (temperature and precipitation) in our models of *P falciparum* and *P vivax* incidence. We found that the effect of temperature on malaria transmission was greater in areas of high mining activity than it was in areas of low mining activity (figure 3B). In high mining areas, temperature was positively associated with incidence of *P falciparum* up to 26·5°C, at which point temperature started to become negatively associated with incidence. At peak temperatures of 26·5°C in high mining areas the effect size for *P falciparum* malaria (2·4 cases per 1000 people [95% CI 1·78–3·06]) was over twice as high as that in low mining areas at the same temperature (1 case per 1000 people [0·68–1·49]). For *P vivax* malaria, the peak in incidence was detected at higher temperatures of 28·1°C, with temperatures above this point having only a slight negative effect on incidence (figure 3B). At 28·1°C, the effect size for *P vivax* malaria in high mining areas was 2·2 cases per 1000 people (95% CI 1·58–2·90) and in low mining areas was 0·9 cases per 1000 people (0·64–1·28). Decreased precipitation was associated with increased malaria incidence only in areas with low levels of mining, whereas the effect of rainfall on malaria transmission in high mining areas was minimal (figure 3D). In low mining areas, dry conditions (2·55 mm rainfall per day) resulted in peak effect size of 2·5 *P falciparum* malaria cases per 1000 people (95% CI 1·98–3·22). For *P vivax* malaria, drier conditions (1·84 mm rainfall per day) resulted in an effect size of 1·7 cases per 1000 people (1·38–2·01).

## Discussion

Environmental degradation caused by mining activity can facilitate the spread of vector-borne diseases, such as malaria, by altering the ecological landscape and increasing exposure to mosquito vectors. Here, we found evidence that mining activity in southern Venezuela was an important determinant of the spatial variation in malaria. Mining activity accounted for 27% of the additional spatial variation of *P falciparum* malaria incidence and 23% of *P vivax* malaria incidence in Bolívar. The ecological changes associated with mining activity, primarily the creation of permanent human-made shallow water bodies, promotes increased survival and abundance of anopheline vectors, such as *A darlingi* and *A albitarsis* sensu lato; the most abundant vectors in Sifontes.^16,33^ Migrant worker populations in gold-mining areas often live in camps and villages with incomplete or no walls, and the highly exophagic (outdoor) biting and resting behaviour of vectors generates high rates of vector–human contact.^14,34^ Hotspots of malaria transmission in Bolívar are maintained by poor access to malaria treatment and by the ecological conditions of mining areas that promote permanent vector breeding habitats and increase vector–human contact rates. Mining activity explained more variation in *P falciparum* malaria incidence than *P vivax* malaria incidence, and the relative risk estimates were greater for *P falciparum* malaria than *P vivax.* In contrast to *P vivax*, which is characterised by multiple relapses of infection,^35^ *P falciparum* malaria is more sensitive to environmental conditions and has been shown to be the predominant circulating parasite in gold-mining areas.^36^ However, it is probable that high human mobility of immunologically naive populations in mining areas, among other factors such as poor access to treatment, contributes to the long-term persistence of *P vivax* in mining hotspots in Bolívar.

The habitat preference of *A darlingi* for fragmented landscapes located at the interface of forested and human-dominated environments has been implicated in patterns of malaria transmission in frontier settlements in the Brazilian Amazon.^37,38^ Greater frequency of forest fringe habitat resulting from deforestation, which *A darlingi* has rapidly adapted to, is associated with increased rates of malaria transmission due to the increased human–vector contact rate that these environments allow for.^37,39-41^ Greater abundances of both *A darlingi* and *A albitarsis* sensu lato have been found in open or deforested areas than in intact forest environments;^42,43^ and in human-dominated landscapes there is also increased biting rates of these vectors.^38^ In addition to increased rates of human exposure *A darlingi* and *A albitarsis* sensu lato, deforested areas are also associated with increased secondary vegetation growth, abundant sunlight, and water pools, all of which provide a favourable habitat for malaria vectors such as *A darlingi* and *A albitarsis* sensu lato.^6,44^ In this study, we found higher *P vivax* malaria incidence, but not *P falciparum* malaria incidence, in deforested areas of Bolívar. This finding is in contrast to previous findings that *P falciparum* is more sensitive to environmental conditions than *P vivax*.^27^ A probable explanation for the positive association between deforestation and *P vivax* in this study is the predominance of *P vivax* in the region, resulting from poor access to treatment and diagnostics. This poor access leads to high malaria exposure and a large proportion of asymptomatic carriers that go undetected, which ultimately sustains transmission rates. This is in contrast to *P falciparum* which does not have such characteristic relapses.

Multiple environmental factors, including climatic factors and environmental degradation, interact in complex socioecological landscapes to determine overall disease risk. Here, we explored how environmental degradation modifies the effect of climate variation on malaria transmission. We found evidence of a synergistic effect of temperature on malaria in areas with high mining activity compared with areas with low mining activity. The positive effect of temperature on malaria transmission was greater in high mining areas of Bolívar than low mining areas. In high mining areas, the effect size for both *P falciparum* and *P vivax* malaria was more than twice as high as the effect size in low mining areas. In areas with reduced forest cover, such as open mining pits, temperatures are typically warmer than nearby forests due to increased sunlight reaching the ground. Warmer temperatures and increased number of stagnant pools of water together establish favourable microclimatic conditions that support *A darlingi* proliferation and accelerate the development of the *Plasmodium* parasite, and subsequent malaria transmission.^9,10,45^ In contrast to the stronger relationship we found between *P vivax* and deforestation in Bolívar than *P falciparum,* the synergistic effect of temperature in high mining areas was stronger for *P falciparum* malaria than for *P vivax.* Previously, *P falciparum* malaria has been shown to be more sensitive to climatic conditions including temperature than *P vivax* because of the characteristic relapsing infections attributed to *P vivax* malaria.^27^ Our results have important implications for targeting treatment, vector control activities, and enhancing surveillance in areas vulnerable to both the effects of a warming climate and land-use changes, such as mining.

Low rainfall was associated with higher malaria transmission in areas of Bolívar of low mining activity than in areas of high mining activity. *A darlingi* is a riverine species;^46^ river levels are more stable during transitional periods after the rainy season and, consequently, mosquito populations are more established during these transitional periods than during the rainy season.^34^ Increased malaria transmission during dry conditions in Bolívar corresponds with other studies in gold mines and in the Amazon region that show low rates of *A darlingi* survival and biting during the rainy season, together with higher parous (reproductive) rates during the rainfall transition period.^14,44,47^ Here, we found a greater effect of rainfall in areas of low mining activity than in areas of high mining activity. As a consequence of greater rainfall in forested compared to degraded areas, the effect of rainfall could have a more destructive effect on larval vector habitats in low mining areas where pools of standing water are less abundant.^48^ Additionally, the higher abundance of mosquitoes in mining areas and deforested landscapes^6,14^ than in forested areas^49^ could protect mosquito populations from the destructive effects of rainfall.

There are a number of limitations to our study, owing to the complex sociopolitical and environmental context. One such limitation was that, although we could account for the presence of mining sites in Bolívar, we could not account for the increased susceptibility of mining communities to an increased risk of malaria.^50^ Also, we could not account for population movements among mining communities. Mine workers in the region, including in other mining hotspots such as in French Guiana, are expected to be highly mobile^34,35^ and national surveillance systems might not record all malaria cases. Further, there might have been under-reporting of malaria cases in our study due to the poor health-care accessibility in mining camps, leading to infections that might not have been reported where they originated.^35^ Another limitation was the minimal temporal variation in our mining data, meaning that we were unable to explore the effects of expansion of mining activities in Bolívar. Also, as a result of the worsening economic situation in Venezuela since 2014, there might have been an increased influx of workers to mining areas, as well as an expansion of the number of areas being deforested for mining activities.^13^ Additionally, the nature of small-scale artisanal mines, which are often established illegally, means that capturing the sheer scale of their effect on malaria risk remains a challenge because they are difficult to regulate and recording them can be difficult. Mining activity, particularly for gold, remains an important barrier to malaria elimination in the Latin American region. Because of lapses in malaria surveillance due to the complex ongoing humanitarian crisis in Venezuela, the quality of the malaria case data might have varied over the study time period, which could have resulted in an under-reporting of cases and missed infections. There was probably substantial national migration across the country during the study period that might have affected underlying population estimates and therefore incidence. Additionally, due to a sparsity of detailed data, we could not account for important changes that affect malaria transmission such as malaria treatment, drug resistance, and relapses. Resistance to antimalarial drugs (including sulfadoxine pyrimethamine and chloroquine), which emerged in 1998, probably prompted changes to treatment regimens including the introduction of artemisinin-based combination therapy in 2004, which could have limited our study as we could not account for these changes and the resistance. Lastly, other factors such as severe shortages of antimalarials, self-treatment, and high relapse and reinfection rates also probably contributed to variation in malaria incidence observed during the study period.

Given the rapidly changing climate, it is important to understand how environmental degradation, such as mining activity, can modify the effect of climatic factors on infectious diseases. Here, we have shown that rapid malaria re-emergence in a vulnerable socioeconomic region was driven by patterns of environmental degradation due to mining that, in turn, modulated the effect of climate on malaria transmission. Mining activity in southern Venezuela was strongly related to hotspots of malaria transmission and environmental degradation from mining activity. Malaria transmission was amplified by the effect of warmer temperatures in areas of high mining activity compared with areas of low mining activity. We have shown that under conditions of socioecological change, it is important to consider how environmental factors interact to determine overall disease risk, using Earth observations to make up shortfalls in ground truth data.

## Supplementary Material

Supplementary Appendix 1

Supplementary Appendix 2

## Data Availability

All data and code that support the findings of this study are available in the Github repository at https://github.com/isabelfletcher/malaria-venezuela.

## Research in context

### Evidence before this study

Environmental degradation, such as deforestation and climate variation, have been shown to affect the transmission and incidence of malaria. We searched PubMed on Sept 9, 2021, for all articles published in English since database inception, using the search terms “malaria”, “mining”, and “climate”. Our literature review showed that although there is evidence implicating mining in malaria transmission, it is predominantly obtained using proxies of mining activity, such as gold prices and gold production. We did not find any study that explored the effect of mining activity on malaria in conjunction with climate variation. It remains unclear whether environmental degradation caused by mining activity modulates the effect of climate on malaria.

### Added value of this study

We explicitly quantify the variation in malaria incidence explained by mining activity, showing that the presence of mining activity explains a large proportion of the unexplained spatial variation in malaria incidence. Mining activity was strongly associated with hotspots of malaria transmission, facilitating the re-emergence of malaria in southern Venezuela. Environmental degradation from mining activity also amplified the effect of warmer temperatures on malaria transmission. The effect of temperature on malaria was greater in areas of high mining activity than it was in areas of low mining activity.

### Implications of all the available evidence

Our findings show that areas of high mining activity are at higher risk of malaria transmission than areas of low mining activity and require targeted malaria control activities, such as increased bednet provisioning and disease surveillance. Our findings highlight the effect of climate variation on malaria among areas of varying degrees of environmental disturbance, an important consideration for health authorities. Areas at risk of both environmental degradation and warming temperatures might require increased targeted interventions and improved surveillance.
