# Supplementary material for: Synergies between environmental degradation and climate variation on malaria re-emergence in southern Venezuela: a spatiotemporal modelling study
Source: Lancet Planet Health. Author manuscript; Available in PMC 2023 Sep 1. (PMC10265648; doi:10.1016/S2542-5196(22)00192-9)
Supplement: Supplementary Appendix 1 [file NIHMS1902390-supplement-Supplementary_Appendix_1.pdf]

THE LANCET
Planetary Health

Supplementary appendix 1

This translation in Spanish was submitted by the authors and we reproduce it as supplied. It has not been peer reviewed. *The Lancet*'s editorial processes have only been applied to the original in English, which should serve as reference for this manuscript.

Supplement to: Fletcher IK, Grillet ME, Moreno JE, et al. Synergies between environmental degradation and climate variation on malaria re-emergence in southern Venezuela: a spatiotemporal modelling study. *Lancet Planet Health* 2022; **6:** e739–48.

Los autores nos proporcionaron esta traducción al español y la reproducimos tal como nos fue entregada. No la hemos revisado. Los procesos editoriales de *The Lancet* se han aplicado únicamente al original en inglés, que debe servir de referencia para este manuscrito.

Background

La degradación ambiental facilita la aparición de enfermedades transmitidas por vectores, tales como la malaria, a través de cambios en el paisaje ecológico que aumenta los hábitats de los vectores y el contacto entre humanos y vectores. Sin embargo, los efectos modificadores de la degradación ambiental sobre las relaciones clima-enfermedad no han sido aun bien explorados. En el presente estudio, investigamos el rápido resurgimiento de la malaria en un área endémica crítica de transmisión al sur de Venezuela y exploramos los efectos sinérgicos de la degradación ambiental causada por la actividad de explotación minera de oro y la variación climática.

Methods

Aplicamos modelos espacio-temporales a la malaria reportada en 46 parroquias del estado Bolívar entre 1996 y 2016, región al sureste de Venezuela, parametrizando dichos modelos con reportes oficiales provenientes del Ministerio de Salud de Venezuela, lo que incluyo datos de población y casos mensuales producidos por *Plasmodium falciparum* y *Plasmodium vivax*. Calculamos la precipitación acumulada y temperatura media utilizando datos a partir del sensor ERA5-Land y utilizamos las anomalías mensuales de la temperatura sobre la superficie del mar como un indicador de eventos de El Niño durante el periodo 1996-2016. La ubicación de sitios mineros sospechosos en el estado Bolívar en 2009, 2017 y 2018 se obtuvieron a partir de la red Amazon Geo-Red de Información Socioambiental Referenciada. Estimamos las medidas de pérdida forestal acumulada y desarrollo urbano por km^2 usando mapas anuales de cobertura terrestre de la Iniciativa de Cambio Climático de la Agencia Espacial Europea entre 1996 y 2016. Modelamos los casos mensuales de malaria por *P. falciparum* y *P. vivax* usando un marco de modelo mixto jerárquico bayesiano. Cuantificamos la variación explicada por la actividad minera, antes de explorar los efectos modificadores de la degradación ambiental en las relaciones entre el clima y la malaria.

Findings

Se observó una reducción del 27 % en la variación espacial adicional no explicada de la incidencia de la malaria por *P. falciparum* y una reducción del 23 % en la malaria por *P. vivax* cuando se incluyó a la minería en los modelos. El efecto de la temperatura sobre la malaria fue mayor en las áreas de intensa minería en comparación a áreas de baja minería,

mientras que el tamaño del efecto de la malaria por *P. falciparum* a temperaturas de 26,5°C (2,4 casos por 1000 personas [95% IC 1,78–3,06]) fue dos veces mayor que el efecto en áreas de baja minería (1 caso por cada 1000 personas [0,68–1,49]).

Interpretation

Se demuestra que los puntos críticos de transmisión de malaria se asocian con la actividad minera en el sur de Venezuela. El aumento de las temperaturas exacerbó la transmisión de la malaria en las zonas mineras, lo que destaca la necesidad de considerar en el futuro cómo la degradación ambiental modula el efecto climático sobre el riesgo de enfermedades, lo que es especialmente importante en áreas sujetas a un rápido aumento de las temperaturas y cambios en el uso de la tierra a nivel mundial. Nuestros hallazgos tienen implicaciones para el progreso hacia la eliminación de la malaria en la región de América Latina. Nuestros hallazgos también son importantes para enfocarse de manera efectiva en programas de tratamiento oportuno y actividades de prevención y control de vectores en áreas mineras con altas tasas de transmisión de malaria.
