# Supplementary material for: Synergies between environmental degradation and climate variation on malaria re-emergence in southern Venezuela: a spatiotemporal modelling study
Source: Lancet Planet Health. Author manuscript; Available in PMC 2023 Sep 1. (PMC10265648; doi:10.1016/S2542-5196(22)00192-9)
Supplement: Supplementary Appendix 2 [file NIHMS1902390-supplement-Supplementary_Appendix_2.pdf]

THE LANCET
Planetary Health

Supplementary appendix 2

This appendix formed part of the original submission and has been peer reviewed. We post it as supplied by the authors.

Supplement to: Fletcher IK, Grillet ME, Moreno JE, et al. Synergies between environmental degradation and climate variation on malaria re-emergence in southern Venezuela: a spatiotemporal modelling study. *Lancet Planet Health* 2022; **6:** e739–48.

Synergies between environmental degradation and climate variation on malaria re-emergence in southern Venezuela: a spatiotemporal modelling study

Appendix

Table of Contents

Climate data 2
Detecting land use changes in Bolívar using remotely sensed land cover data 2
Figure S1. Parishes in Bolívar....3
Figure S2. Mining activity in Venezuela. 4
Figure S3. Land use change in Bolívar, southern Venezuela. 6
Table S1. Model adequacy of linear and non-linear models of malaria incidence in Bolívar. 6
Figure S4. Associations between El Niño and climate conditions in Bolívar. 7
Figure S5. Variation in malaria incidence explained by mining activity....8
Figure S6. Influence of El Niño on malaria incidence at varying monthly lags. 9
Table S2. Land cover classifications summarised from the original ESA CCI land cover classes. 9
Figure S7. Bivariate relationship between deforestation, mining, and urbanization in Bolívar. 10
Figure S8. Environmental and socio-economic drivers in linear models of malaria in Bolívar state....11
Figure S9. Influence of climate variation on malaria incidence in Bolívar state. 12
References....13

Climate data

Monthly estimates for mean temperature and precipitation in each parish in Bolívar, obtained from the ERA5-Land dataset[1] were included into the models of malaria incidence by taking spatially aggregated estimates at the parish level (admin 3). We captured the lagged effect of climate on malaria transmission, which results from the time required for completion of mosquito and parasite life cycles, and the time between malaria diagnosis and reporting[2,3], by averaging climate conditions of the previous three months for each parish. Monthly sea-surface temperature anomalies for the Niño 3·4 region, an index of the El Niño Southern Oscillation (ENSO), a major climatic phenomenon affecting the northern coast of South America were obtained from the National Oceanic and Atmospheric Administration (NOAA). The Niño 3·4 index has previously been linked to the interannual variability of malaria across Venezuela[4] and brings warm and mostly dry conditions to Bolívar (Figure S4). The Niño 3·4 anomalies were lagged by eight months (Figure S6), which is in broad agreement with previous studies that have associated seasonal malaria with SST anomalies in the Niño 3·4 region with a delay of 9-12 months[4].

In all our models, climate variables (temperature and precipitation) were included as nonlinear terms, by using a random walk of order 1, using a smooth term to represent nonlinearity. High temperatures above 30°C can limit mosquito and parasite survival and development, and large amounts of rainfall can flush out mosquito larval habitats, leading to a decrease in malaria transmission[5–7]. In our study, we found a similar non-linear relationship between malaria and temperature, and dry conditions were associated with increased malaria risk (Figure S9). We also tested a linear model for *P. falciparum* and *P. vivax* malaria, which showed no significant association for temperature (Figure S8) and produced a worse model fit that our non-linear model as measured by an increase in DIC and WAIC values (Table S1).

Detecting land use changes in Bolívar using remotely sensed land cover data

In order to investigate land use changes, specifically forest loss and urbanization, in Bolívar between 1996-2016 we obtained annual land cover maps of 300 m spatial resolution from the European Space Agency (ESA) Climate Change Initiative (CCI) (https://www.esa-landcover-cci.org/). We identified annual land cover changes that occurred between 1996-2016 for each parish in Bolívar by first reclassifying the land cover maps. We aggregated the CCI land cover classes into broader categories of interest that included urban areas and forested areas (Table S2). We then calculated the number of grid cells, per parish 1996-2016, that were classed as forest cover, and for each year identified where forest cover had decreased compared to the previous year. These values were summed across each parish in Bolívar to give a measure of cumulative forest loss (Figure S3A). We repeated the same process for urban areas to obtain a yearly measure of urbanization for each parish 1996-2016 (Figure S3B). The relationship between deforestation and urbanization, with mining activity is shown in Figure S7.

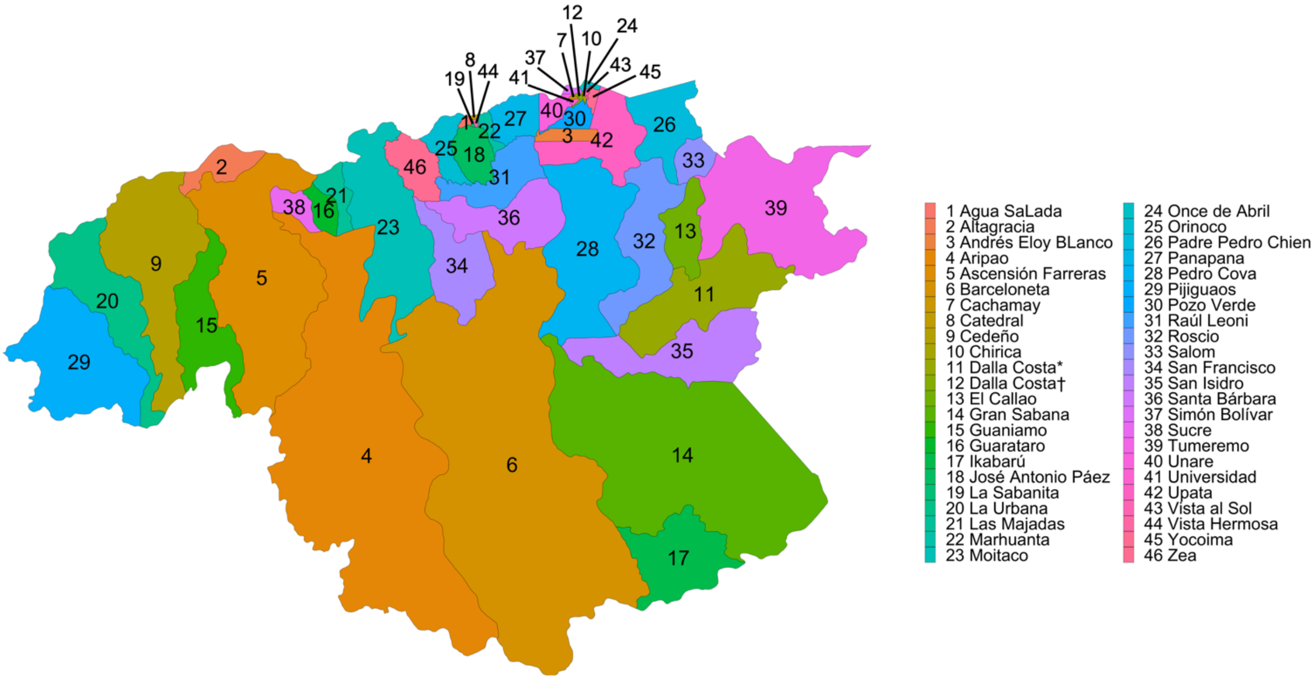

Figure S1. Parishes in Bolívar.

Location of 46 parishes in Bolívar state, Venezuela. *Dalla Costa parish, Sifontes municipality. †Dalla Costa parish, Caroní municipality.

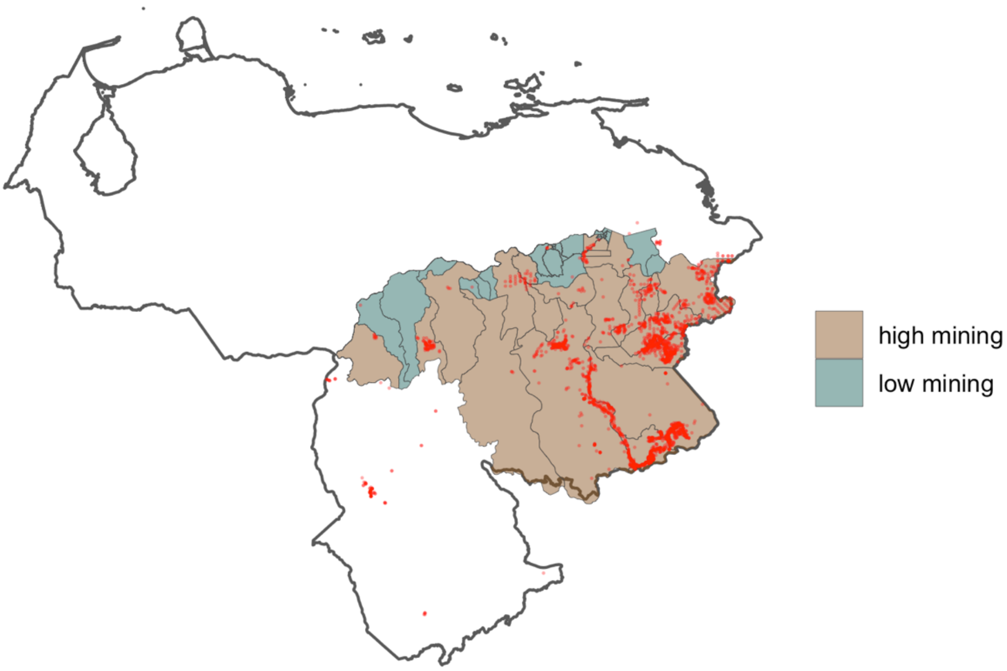

Figure S2. Mining activity in Venezuela.

Location of mines (red) in Venezuela (n = 2,561) of which the majority are in Bolívar state (n = 2, 460) (inset map). Parishes in Bolívar are classified as having high (brown, n = 22) and low (green, n = 24) levels of mining activity.

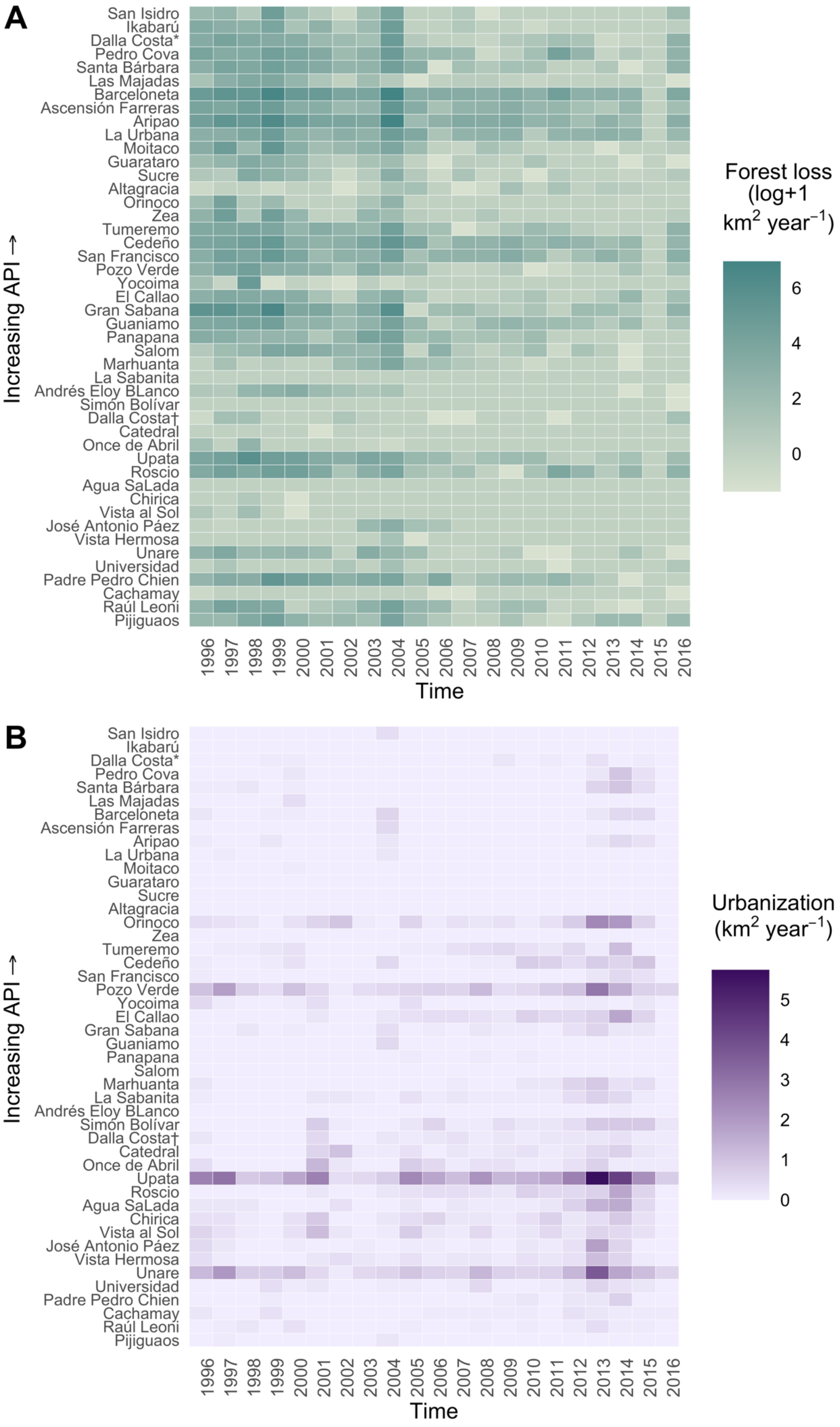
A
San Isidro
Ikabarú
Dalla Costa*
Pedro Cova
Santa Bárbara
Las Majadas
Barceloneta
Ascensión Farreras
Aripao
La Urbana
Moitaco
Guarataro
Sucre
Altagracia
Orinoco
Zea
Tumeremo
Cedeño
San Francisco
Pozo Verde
Yocoima
El Callao
Gran Sabana
Guaniamo
Panapana
Salom
Marhuanta
La Sabanita
Andrés Eloy BLanco
Simón Bolívar
Dalla Costa†
Catedral
Once de Abril
Upata
Roscio
Agua SaLada
Chirica
Vista al Sol
José Antonio Páez
Vista Hermosa
Unare
Universidad
Padre Pedro Chien
Cachamay
Raúl Leoni
Pijiguaos
Increasing API →
1996 1997 1998 1999 2000 2001 2002 2003 2004 2005 2006 2007 2008 2009 2010 2011 2012 2013 2014 2015 2016
Time
Forest loss (log+1 km^2 year^{-1})
6
4
2
0
B
Increasing API →
Time
Urbanization (km^2 year^{-1})
5
4
3
2
1
0

Figure S3. Land use change in Bolívar, southern Venezuela.

A) Annual decrease (log +1 km^2) 1996-2016 in forest cover identified from satellite land cover maps for the 46 parishes of Bolívar state. Parishes are ordered by annual parasite incidence (API), with those at the top representing areas with the highest malaria incidence and those at the bottom with the lowest recorded malaria incidence. B) Annual increase (km^2) 1996-2016 in urban cover identified from satellite land cover maps for the 46 parishes of Bolívar state. Parishes are ordered by annual parasite incidence (API), with those at the top representing areas with the highest malaria incidence and those at the bottom with the lowest recorded malaria incidence. *Dalla Costa parish, Sifontes municipality. †Dalla Costa parish, Caroní municipality.

Table S1. Model adequacy of linear and non-linear models of malaria incidence in Bolívar.

Deviance information criterion (DIC) and Watanabe-Akaike information criterion (WAIC) for spatiotemporal models of monthly *P. falciparum* and *P. vivax* malaria incidence, $\log(\rho_{st})$ in Bolívar 1996-2016 that included the linear effect of climate and the non-linear effect of climate and its interaction with mining, $f(x1_m) + f(x2_m)$. Models also included the impact of deforestation, urbanization, and El Niño (expressed as a combination of spatiotemporal covariates, ($\Sigma\beta_i x_{ist}$) and random effects for month (m_t) and year (a_t), to account for seasonality, interannual variability and spatial dependency structures ($\upsilon_s + v_s$).

Model formula	Parasite	DIC	WAIC
Linear climate model	*P. falciparum*	40825.79	40858.83
$\log(\rho_{st}) = \alpha + \Sigma\beta_i x_{ist} + m_t + a_t + \upsilon_s + v_s$	*P. vivax*	61689.81	61729.14
Non-linear climate model, including an interaction between climate and level of mining	*P. falciparum*	40686.93	40729.93
$\log(\rho_{st}) = \alpha + \Sigma\beta_i x_{ist} + f(x1_m) + f(x2_m) + m_t + a_t + \upsilon_s + v_s$	*P. vivax*	61561.05	61621.21

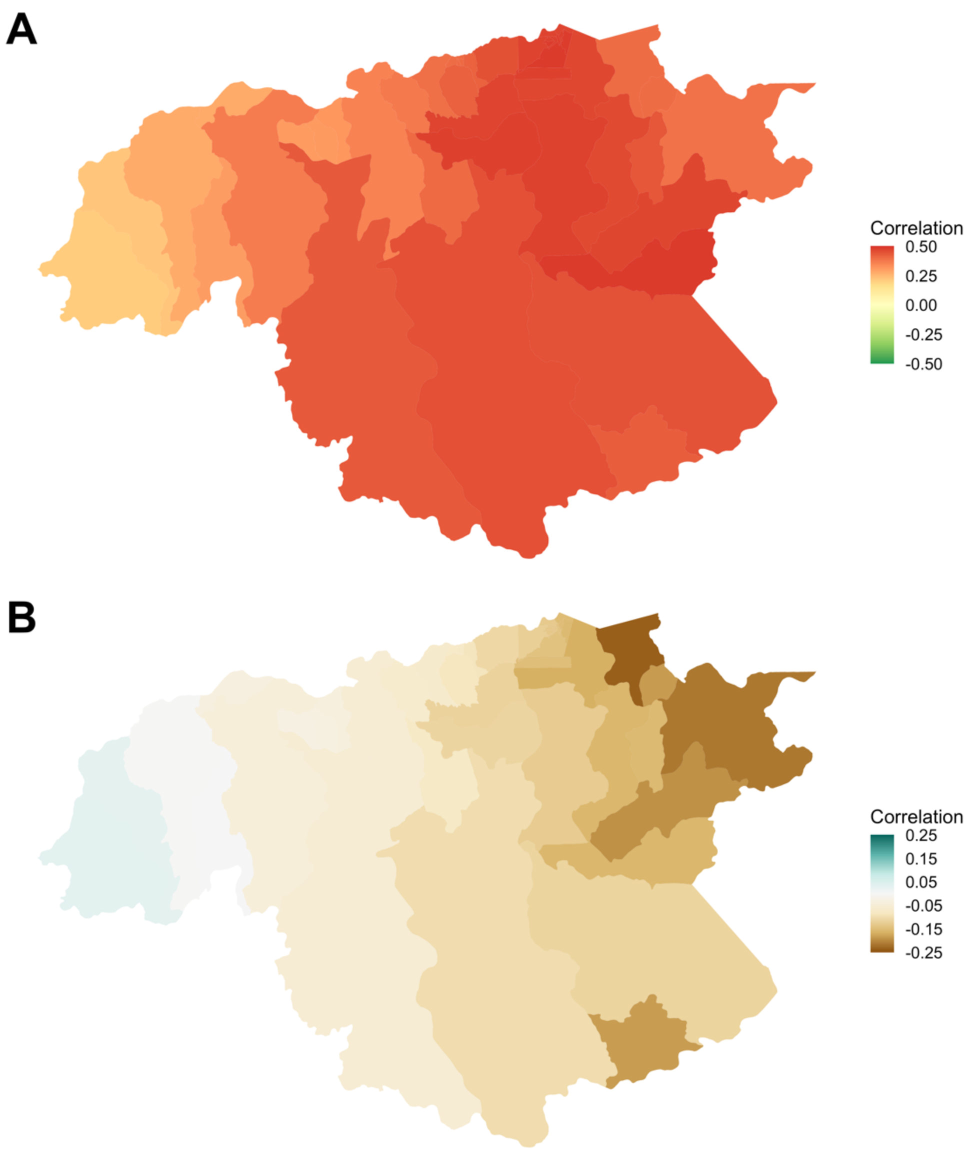

Figure S4. Associations between El Niño and climate conditions in Bolívar.

Pearson correlation coefficients between the Niño 3.4 index and A) mean temperatures (°C) and B) mean precipitation (mm/day) between 1996-2016 in Bolívar, southern Venezuela. Climate data are aggregated to the parish level.

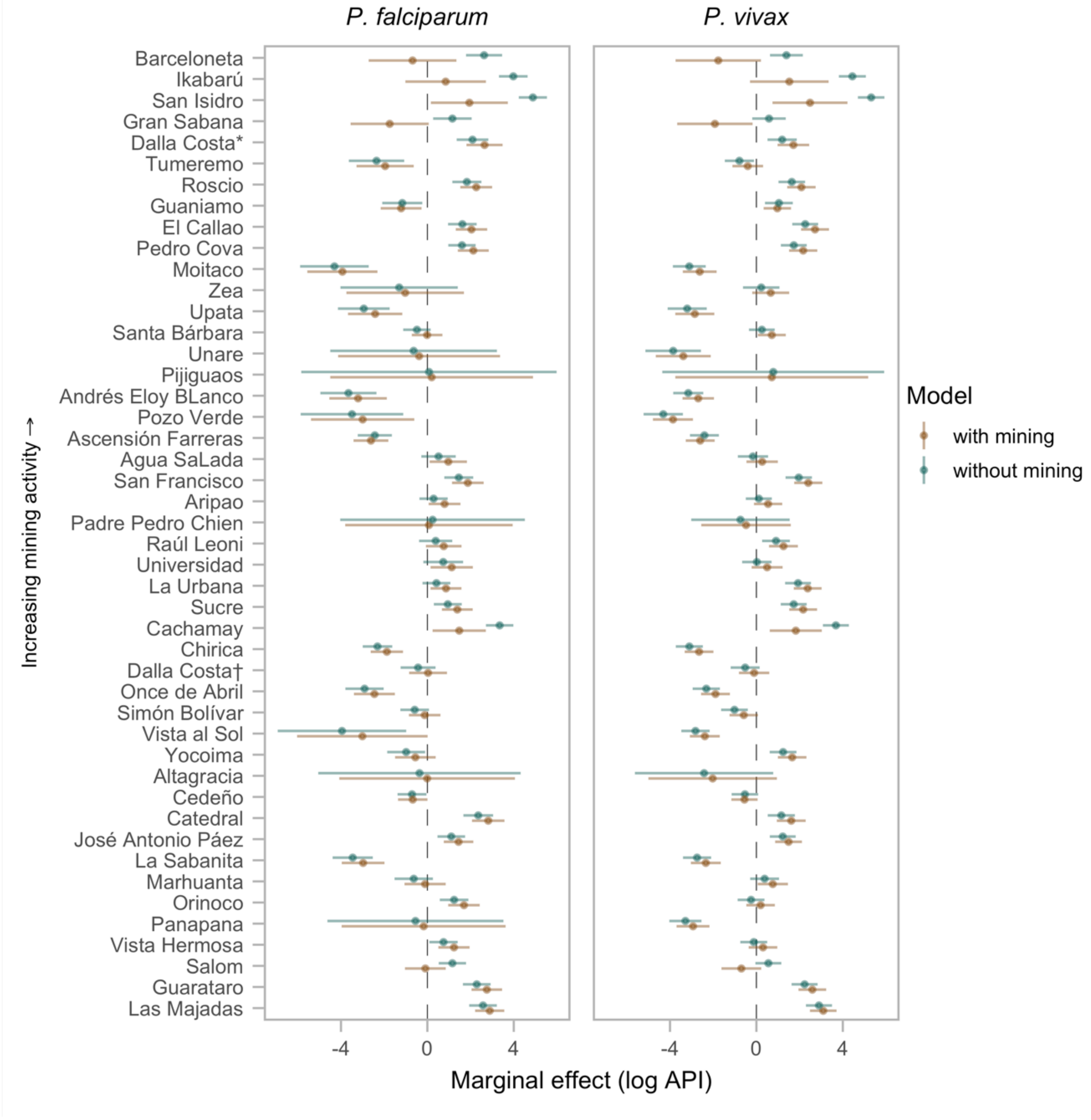

Figure S5. Variation in malaria incidence explained by mining activity.

Marginal effect (mean and 95% credible intervals of the spatial random effect) of log annual parasite incidence (API), of spatiotemporal models for *P. falciparum* (left panel) and *P. vivax* (right panel) malaria that exclude (light blue) and include (dark blue) mining activity across Bolívar as a covariate. A reduction in mean estimate towards zero indicates where mining activity explains the spatial variation in malaria incidence. The model also included the impact of deforestation, urbanization, El Niño and an interaction term between level of mining and nonlinear functions of temperature and rainfall, and random effects, to account for seasonality, interannual variability and spatial dependency structures. *Dalla Costa parish, Sifontes municipality. †Dalla Costa parish, Caroní municipality.

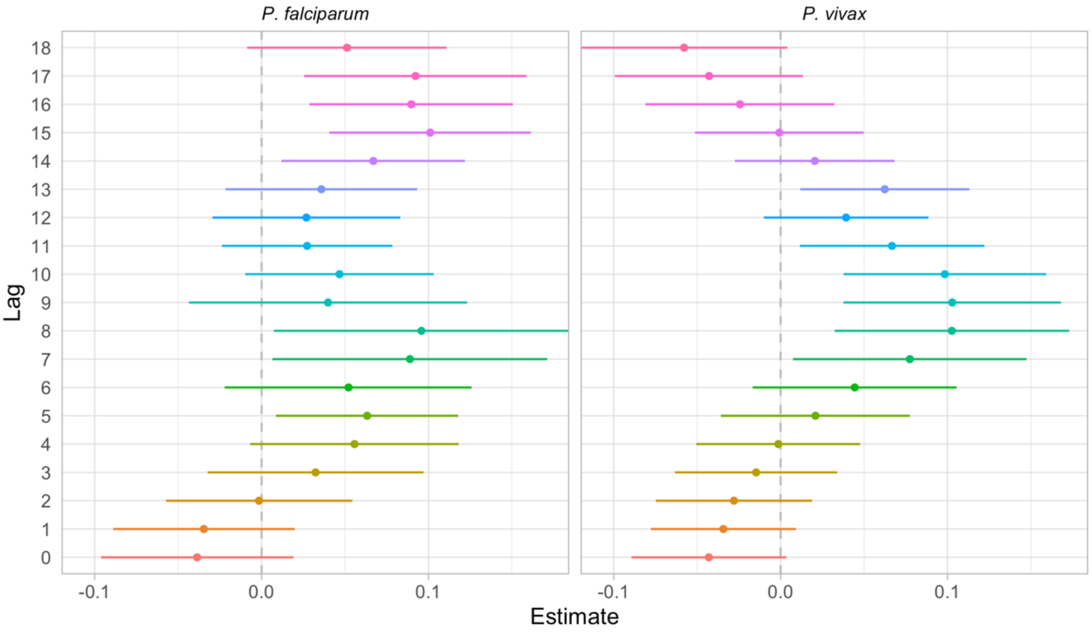

Figure S6. Influence of El Niño on malaria incidence at varying monthly lags.

Posterior mean estimates and 95% credible intervals for spatiotemporal models of *P. falciparum* (left panel) and *P. vivax* (right panel) malaria in Bolívar state. The model included the Niño 3.4 index lagged from zero to 18 months, and also included an interaction term between high and low levels of mining and nonlinear functions of temperature and rainfall. The model also included the impact of deforestation, urbanization and random effects, to account for seasonality, interannual variability and spatial dependency structures.

Table S2. Land cover classifications summarised from the original ESA CCI land cover classes.

Seven classes were aggregated to forest/tree cover and a single class defined as urban. The change in these land cover classes over time were then used to extract variables of deforestation and urbanization.

Value	Label	Revalued class
50	Tree cover, broadleaved, evergreen, closed to open (>15%)	
60	Tree cover, broadleaved, deciduous, closed to open (>15%)	
70	Tree cover, needleleaved, evergreen, closed to open (>15%)	
80	Tree cover, needleleaved, deciduous, closed to open (>15%)	Forest/tree cover
90	Tree cover, mixed leaf type (broadleaved and needleleaved)	
100	Mosaic tree and shrub (>50%) / herbaceous cover (<50%)	
110	Mosaic herbaceous cover (>50%) / tree and shrub (<50%)	
190	Urban areas	Urban

A

B

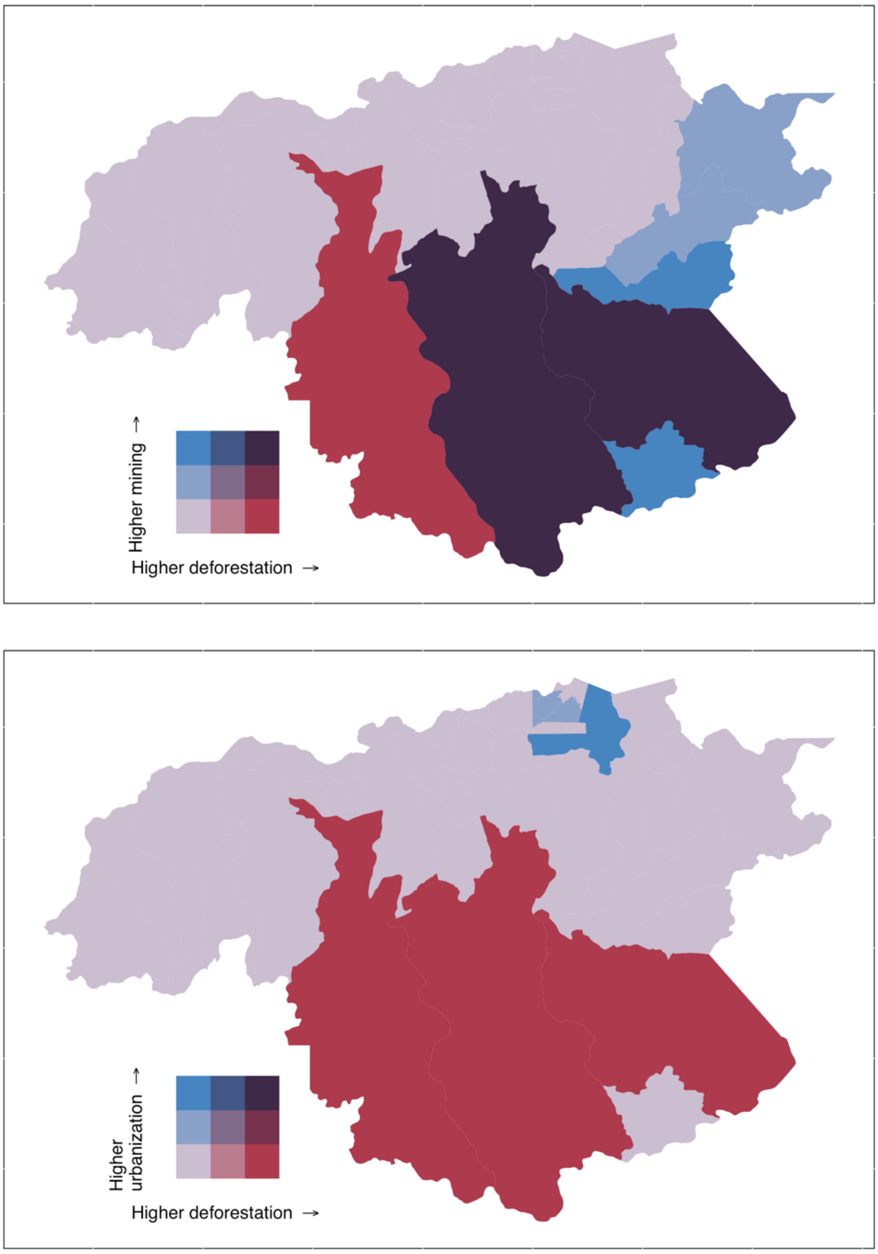

Figure S7. Bivariate relationship between deforestation, mining, and urbanization in Bolívar.

A) Relationship between mining activity and forest loss between 1996-2016 across Bolívar. Dark purple colours represent parishes with both high levels of mining and high levels of deforestation, whilst pale colours represent areas with minimal mining activity and low deforestation. B) Relationship between urbanization and forest loss between 1996-2016 across Bolívar. Dark purple colours represent parishes with both high levels of urbanization and high levels of deforestation, whilst pale colours represent areas with low urbanization and low deforestation. Data shown is aggregated to the parish level.

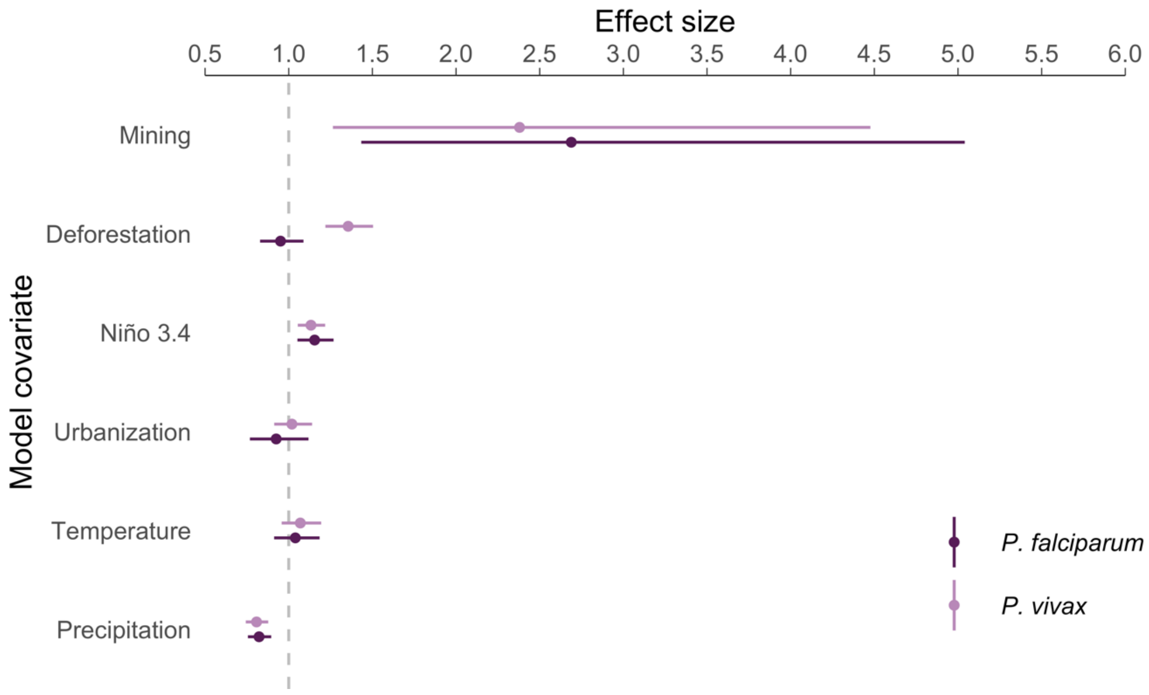

Figure S8. Environmental and socio-economic drivers in linear models of malaria in Bolívar state.

Effect size and 95% credible intervals for spatiotemporal models of *P. falciparum* (purple bars) and *P. vivax* (pink bars) malaria incidence. Models accounted for the linear effect of climate (temperature and precipitation), and included random effects, to account for seasonality, interannual variability and spatial dependency structures.

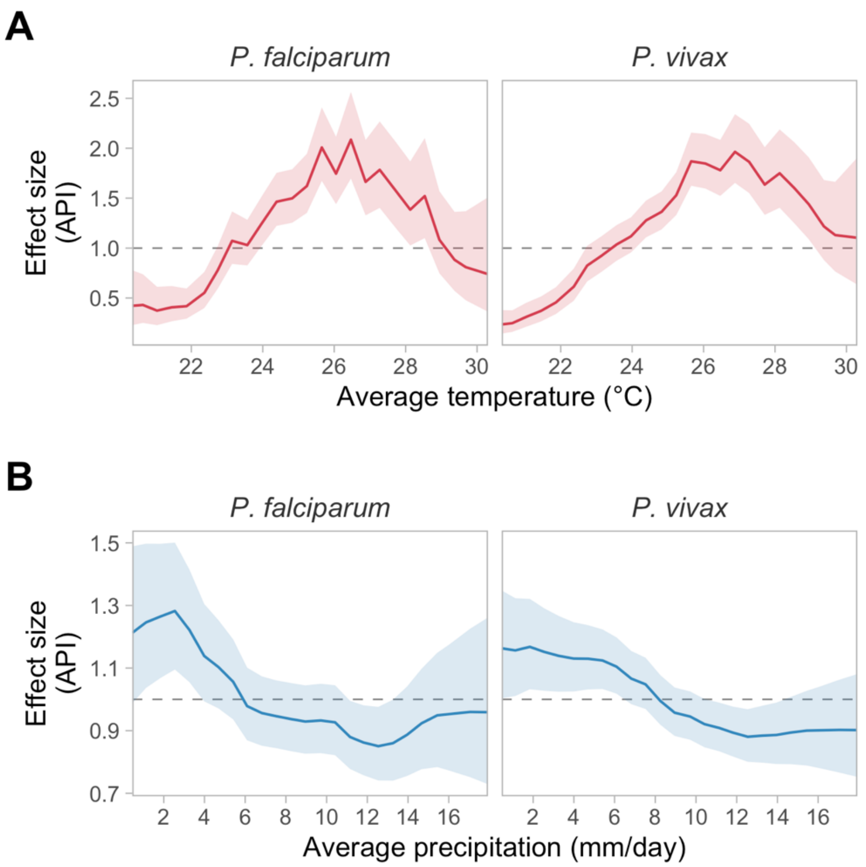

Figure S9. Influence of climate variation on malaria incidence in Bolívar state.

Effect size (API) of the relationship between mean temperature (A) and precipitation (B) and *P. falciparum* and *P. vivax* malaria. The model included nonlinear functions of temperature and rainfall and included the impact of mining, deforestation, urbanization, El Niño and random effects, to account for seasonality, interannual variability and spatial dependency structures.

References

1. The Copernicus Global Land Service. (2019). Available at: https://land.copernicus.eu/global/. (Accessed: 15th January 2019)
2. Ikeda, T. *et al.* Seasonally lagged effects of climatic factors on malaria incidence in South Africa. *Sci. Rep.* **7**, 2458 (2017).
3. Laneri, K., Cabella, B., Prado, P. I., Mendes Coutinho, R. & Kraenkel, R. A. Climate drivers of malaria at its southern fringe in the Americas. *PLoS One* **14**, e0219249 (2019).
4. Grillet, M. E., El Souki, M., Laguna, F. & León, J. R. The periodicity of Plasmodium vivax and Plasmodium falciparum in Venezuela. *Acta Trop.* **129**, 52–60 (2014).
5. Mordecai, E. A. *et al.* Optimal temperature for malaria transmission is dramatically lower than previously predicted. *Ecol. Lett.* **16**, 22–30 (2013).
6. Shapiro, L. L. M., Whitehead, S. A. & Thomas, M. B. Quantifying the effects of temperature on mosquito and parasite traits that determine the transmission potential of human malaria. *PLoS Biol.* **15**, e2003489 (2017).
7. Wolfarth-Couto, B., Silva, R. A. da & Filizola, N. Variability in malaria cases and the association with rainfall and rivers water levels in Amazonas State, Brazil. *Cad. Saude Publica* **35**, e00020218 (2019).
